# Quantitative evaluation of the effect of hyperlipidemia on carotid artery stiffness in patients with type 2 diabetes mellitus using shear wave elastography

**DOI:** 10.1097/MD.0000000000050829

**Published:** 2026-09-25

**Authors:** Yuanyuan Liu, Xi Yang, Xuemeihui Ma, Dan Lv, Guangsen Li

**Affiliations:** a Department of Ultrasound, The Second Affiliated Hospital of Dalian Medical University, Dalian, Liaoning, China.

**Keywords:** carotid artery stiffness, hyperlipidemia, shear wave elastography, type 2 diabetes

## Abstract

Type 2 diabetes mellitus (T2DM) is a major risk factor for atherosclerotic cardiovascular disease (CVD), and hyperlipidemia (HLD) frequently coexists. Shear wave elastography (SWE) quantifies carotid stiffness and may improve CVD risk stratification. To evaluate the impact of HLD on carotid stiffness in T2DM using SWE. We conducted a cross-sectional study of 112 adults with T2DM: simple diabetes (Group B, n = 52) and T2DM with HLD (Group C, n = 60): and 55 healthy controls (Group A). Demographic, clinical, and biochemical data were collected. Conventional ultrasound measured carotid intima-media thickness, diastolic diameter, systolic diameter, and peak systolic velocity. Arterial distensibility and the stiffness coefficient were calculated. SWE provided maximum, mean, and minimum elastic moduli. Pearson correlation tested associations between SWE parameters and risk factors; multivariable linear regression identified independent determinants of SWE values. Compared with controls, Groups B and C had higher fasting glucose, glycated hemoglobin, total cholesterol (TC), triglycerides (TG), and LDL-C (all *P *< .05); TC, TG, and LDL-C were higher in Group C than Group B (all *P *< .05). high-density lipoprotein cholesterol (HDL-C) were lower in both Group B and Group C compared to Group A (both *P *< .05), and were also lower in Group C than in Group B (*P *< .05). Mean elastic modulus, maximum elastic modulus, and minimum elastic modulus were greater in Groups B and C than in controls and were highest in Group C (all *P *< .05). SWE parameters correlated positively with carotid intima-media thickness, TC, TG, and LDL-C, and negatively correlated with HDL-C (all *P *< .05). In multivariable models, hyperlipidemia: driven primarily by elevated TC, TG, and LDL-C and reduced HDL-C: was independently associated with increased SWE values in T2DM (all *P *< .05). HLD exacerbates carotid atherosclerosis in T2DM. SWE is a noninvasive, quantitative, and efficient method for assessing carotid stiffness and cardiovascular risk in T2DM, particularly among patients with concurrent HLD.

## 1. Introduction

Type 2 diabetes mellitus (T2DM) is a chronic metabolic disorder characterized by abnormally elevated blood glucose levels. Recent global estimates suggest that the prevalence of T2DM is approximately 10.5% among individuals aged 20 to 79 years.^[1]^ According to the American Diabetes Association, 60% to 70% of T2DM patients also suffer from hyperlipidemia (HLD).^[2]^ The metabolic disturbances caused by high glucose levels in the presence of HLD often lead to the production of excess harmful byproducts. For example, the overactivation of the sorbitol pathway increases the activity and content of aldose reductase, which contributes to insulin resistance, extensive oxidative stress, and elevated inflammatory cytokines, thereby damaging the vascular system.^[3,4]^ T2DM itself is a well-established risk factor for early carotid atherosclerosis. Furthermore, HLD exacerbates glycotoxicity and synergistically promotes atherosclerosis in conjunction with dyslipidemia.^[5]^ HLD is commonly characterized by elevated triglycerides (TG) and/or total cholesterol (TC) levels in the plasma. Increasing evidence indicates that elevated cholesterol levels in triglyceride-rich lipoproteins are a significant risk factor for atherosclerosis and contribute to the acceleration of cardiovascular disease development.^[6]^

Cardiovascular disease typically manifests in its later stages of atherosclerosis, although there is often a prolonged asymptomatic period beforehand.^[7]^ Changes in subclinical atherosclerosis are reflected in the arterial wall, including the thickening of the intima-media and a decrease in vascular elasticity.^[8]^ Cervical femoral pulse wave velocity is widely regarded as the gold standard for assessing arterial stiffness in clinical practice. However, cervical femoral pulse wave velocity provides only an approximate estimate of pulse wave velocity from the aortic arch to the femoral artery and does not allow for localization of specific vascular segments.^[9]^ Although being an early morphological indicator of atherosclerosis, carotid intima-media thickness (CIMT) is preceded by functional alterations in the vascular system.^[10]^ Shear wave elastography (SWE) is an emerging ultrasound technique that provides direct, real-time, and noninvasive measurements of the longitudinal stiffness of arterial blood vessels. It can quantitatively assess the absolute value of tissue stiffness and evaluate changes in carotid artery stiffness.^[11]^ SWE has been widely applied in the evaluation of superficial organs such as the thyroid, liver, and breast, proving highly effective in diagnosing lesions such as fibrosis and tumors.^[12–14]^ In recent years, this technology has been increasingly utilized for vascular assessments.

The purpose of this study is to quantitatively evaluate the effect of HLD on carotid artery stiffness in patients with T2DM using SWE, thereby assessing subclinical atherosclerosis through quantitative measures.

## 2. Materials and methods

### 2.1. Study population

112 patients with T2DM who were admitted to our hospital between March 2024 and March 2025 were selected for the study. The patients were divided into 2 groups. Group B (simple diabetes group) included 52 patients, comprising 25 females and 27 males, with an average disease duration of 45.0 ± 6.8 months. Group C (T2DM with HLD) included 60 patients, consisting of 30 females and 30 males, with an average disease duration of 46.8 ± 6.6 months. Additionally, 55 healthy individuals were selected to form Group A (control group), which included 27 males and 28 females. The inclusion criteria were as follows: Participants met the diagnostic criteria for T2DM as outlined in the 2023 guidelines for the definition, classification, diagnosis, screening, and prevention of diabetes.^[15]^ Patients with HLD were defined as those with fasting serum TC levels exceeding 5.72 mmol/L or triglyceride levels exceeding 1.7 mmol/L.^[16]^ Every subject willingly consented to participate in the experiment after being informed about it. No significant plaque formation was observed in the left carotid artery as assessed by ultrasound. The exclusion criteria were as follows: Patients with previously diagnosed cardiovascular or cerebrovascular conditions, including heart failure, carotid stenosis, coronary artery disease, hypertension, and stroke. Patients with severe diseases such as immunodeficiency disorders or malignant tumors. Patients with abnormal kidney function. Patients with carotid artery stiffness changes attributable to other medical conditions.

This study was approved by the Medical Ethics Committee of the Second Affiliated Hospital of Dalian Medical University (ethics approval number: KY2025-335-01). All participants were fully informed about the study details and provided written informed consent.

### 2.2. General information and biochemical data

General information, including gender, age, disease duration, body mass index (BMI), blood pressure, heart rate, and smoking status, was collected from all participants. Venous blood samples were drawn after a 12-hour overnight fast (including water deprivation). Biochemical markers, including fasting blood glucose (FBG), glycated hemoglobin (HbA1c), TC, TG, low-density lipoprotein cholesterol (LDL-C), and high-density lipoprotein cholesterol (HDL-C), were measured using a fully automated biochemical analyzer.

### 2.3. Conventional ultrasound measurement parameters

A Mindray ultrasound diagnostic system, equipped with an L4-15WU linear array probe (frequency range: 10–12 MHz), was used to perform routine carotid artery ultrasound examinations on all participants. M-mode ultrasound was employed to measure the maximum systolic diameter and diastolic diameter (Dd) of the carotid artery at 1.0 to 1.5 cm distal to the left common carotid artery bulb. Two-dimensional ultrasound was used to assess CIMT, while Doppler ultrasound was used to quantify peak systolic velocity. All parameters were measured in triplicate, and the mean values were calculated. Using these data and standard formulas, the arterial distensibility (arterial distensibility [*ΔD*], *ΔD* = systolic diameter-Dd) and wall stiffness coefficient (*β*, *β*=ln(SBP/DBP)/[(*ΔD*)/Dd]) were derived. Final values were derived as the mean of the 3 replicate measurements.

### 2.4. Shear wave elasticity imaging procedure

Using the same ultrasound diagnostic system, the depth and gain settings were optimized to ensure clear, high-quality 2-dimensional images. The system was then switched to the SWE mode while maintaining the probe’s position. To minimize motion artifacts, participants were instructed to avoid swallowing or coughing, breathe calmly, and hold their breath after stabilizing their respiration. The probe was adjusted to visualize carotid artery segments where the intima-media layer displayed clear, homogeneous, color-coded signals in both the anterior and posterior walls. To ensure image quality, only segments with well-defined borders and consistent color-coding across the vessel walls were selected for measurement. Frames with a motion stability index of 4 or 5 stars (provided by the ultrasound system) were accepted. End-diastolic images were frozen and stored. This process was repeated 3 times to capture 3 optimal images, ensuring that the same segment was consistently measured across all repetitions. The quality of each image was visually assessed based on the clarity of the intima-media interface, absence of motion artifacts, and uniformity of the color-coded signals. Using the system’s measurement software, a circular region of interest with a 1 mm diameter was positioned at the central anterior wall intima, aligned parallel to the carotid artery axis. Measurements were taken at 2 mm intervals along a 10 mm segment. For each image, the average, minimum, and maximum Young’s modulus values were calculated from 10 sequential measurements. The mean elastic modulus (MEmean), minimum elastic modulus (MEmin), and maximum elastic modulus (MEmax) were then derived by averaging the values across the 3 stored images.

Forty participants were randomly selected from the study cohort. Two blinded ultrasound physicians independently measured left carotid artery stiffness parameters using identical protocols. After a 7 day interval, the same parameters were reassessed in these participants by 1 physician to determine interobserver and intraobserver reliability. Intraclass correlation coefficients with 95% confidence intervals were calculated to assess interobserver and intraobserver variability.

### 2.5. Statistical analysis

Statistical analyses were performed using SPSS 27.0 (IBM Corp., Armonk, NY). Normally distributed quantitative data are presented as mean ± standard deviation (*__x ± s*). Chi-square tests were used for categorical variables, and 1-way ANOVA with post hoc Tukey’s test was applied for continuous variables. Inter-group comparisons were conducted using independent samples *t* tests. Pearson correlation analysis assessed relationships between SWE parameters and CIMT, TC, TG, LDL-C and HDL-C. The relationship between HLD and SWE parameters of carotid artery in patients with T2DM was observed by multiple linear stepwise regression analysis with MEmax, MEmean, and MEmin as the dependent variables and gender, age, disease duration, smoking status, BMI, SBP, HbA1c, TC, TG, LDL-C and HDL-C as the independent variables, respectively.

## 3. Results

### 3.1. Participants characteristics

No statistically significant differences were observed in gender, age, disease duration, BMI, blood pressure, heart rate, or smoking status among the 3 groups (all *P* > .05). (see Table 1).

**Table 1:** Comparison of general clinical parameters among the 3 groups.

Parameter	Group A(n* *= 55)	Group B(n = 52)	Group C(n = 60)	*F*/*X *^2^/*t* value	*p* value
Gender (M/F)	27/28	27/25	30/30	0.089	.956
Age (yr)	57.8 ± 7.0	57.8 ± 9.3	56.2 ± 8.5	0.682	.507
Duration(mo)	/	45.0 ± 6.8	46.8 ± 6.6	−1.352	.179
BMI (Kg/m^2^)	24.4 ± 2.8	25.1 ± 3.8	25.3 ± 2.8	1.088	.339
SBP (mm Hg)	114.7 ± 11.4	116.5 ± 11.4	116.6 ± 11.2	0.481	.619
DBP (mm Hg)	71.2 ± 5.3	70.7 ± 5.6	71.8 ± 6.3	0.514	.599
HR (bpm)	74.2 ± 4.8	73.5 ± 4.3	74.8 ± 3.7	1.270	.284
Current smoker, n (%)	10 (18.2)	12 (23.1)	14 (23.3)	0.554	.758

1 mm Hg = 0.133 kPa. “*” indicates comparison with Group A, *P *< .05. “#” indicate.

### 3.2. Biochemical indicators

The biochemical indicators, including FBG, HbA1c, TC, TG, LDL-C, were significantly higher than those in Group A (all *P *< .05). Additionally, TC, TG, and LDL-C in Group C were significantly higher than those in Group B (all *P* < .05). HDL-C were significantly lower in both Group B and Group C compared to Group A (both *P *< .05), and were also significantly lower in Group C than in Group B (*P *< .05). (see Table 2).

**Table 2:** Comparison of biochemical indicators among the 3 groups (Mean ± SD).

Parameter	Group A (n = 55)	Group B(n = 52)	Group C(n = 60)	*F* value	*p* value
FBG (mmol/L)	4.99 ± 0.69	8.27 ± 1.91*	8.42 ± 1.69*	151.255	<.001
HbA1c (%)	5.11 ± 0.58	8.92 ± 1.43*	8.84 ± 1.85*	239.572	<.001
TC (mmol/L)	3.54 ± 0.56	4.04 ± 0.56*	6.12 ± 0.69*^,^#	285.551	<.001
TG (mmol/L)	0.83 ± 0.23	1.17 ± 0.30*	2.48 ± 0.84*^,^#	113.036	<.001
LDL-C (mmol/L)	1.80 ± 0.48	2.27 ± 0.51*	3.23 ± 0.88*^,^#	60.103	<.001
HDL-C (mmol/L)	1.46 ± 0.18	1.20 ± 0.19*	1.03 ± 0.22*^,^#	68.088	<.001

“*” indicates comparison with Group A, *P *< .05.

“#” indicates comparison with Group B, *P* < .05.

### 3.3. Conventional ultrasound parameters

Both Group B and Group C were higher CIMT and *β* values compared to Group A (both *P *< .05). Although Group C was slightly higher CIMT and *β* values than Group B, the results did not show statistical differences (both *P *> .05). (See Table 3).

**Table 3:** Comparison of conventional carotid ultrasound parameters among the 3 groups (Mean ± SD).

Parameter	Group A(n = 55)	Group B(n = 52)	Group C(n = 60)	*F* value	*p* value
Ds (mm)	6.90 ± 0.38	6.93 ± 0.31	6.94 ± 0.29	0.222	.801
Dd (mm)	6.35 ± 0.39	6.40 ± 0.29	6.43 ± 0.28	0.778	.462
*ΔD* (mm)	0.54 ± 0.14	0.52 ± 0.14	0.50 ± 0.15	0.841	.433
PSV (cm/s)	69.28 ± 9.71	69.59 ± 9.84	71.02 ± 8.13	0.587	.557
IMT (mm)	0.64 ± 0.24	0.89 ± 0.22*	0.93 ± 0.22*	25.364	<.001
*β*	5.80 ± 1.36	6.39 ± 1.58*	6.47 ± 1.36*	3.615	.029

“*” indicates comparison with Group A, *P *< .05.

### 3.4. SWE parameters

The MEmean, MEmax, and MEmin of Groups B and C were significantly higher than those of Group A (all *P *< .05). Furthermore, Group C’s SWE values were substantially greater than those of Group B (all *P *< .05). (See Table 4 and Fig. 1).

**Table 4:** Comparison of carotid SWE parameters among the 3 groups (Mean ± SD).

Parameter	Group A (n = 55)	Group B (n = 52)	Group C (n = 60)	*F* value	*P* value
MEmax (kPa)	72.04 ± 10.36	85.62 ± 10.95*	100.73 ± 10.88*^,^#	102.672	<.001
MEmean(kPa)	57.80 ± 10.13	68.55 ± 11.47*	86.65 ± 9.60*^,^#	113.616	<.001
MEmin(kPa)	46.83 ± 8.51	56.55 ± 9.35*	69.61 ± 9.71*^,^#	88.553	<.001

Note: “*” indicates comparison with Group A, *P *< .05.

“ #” indicates comparison with Group B, *P* < .05.

**Figure 1.**
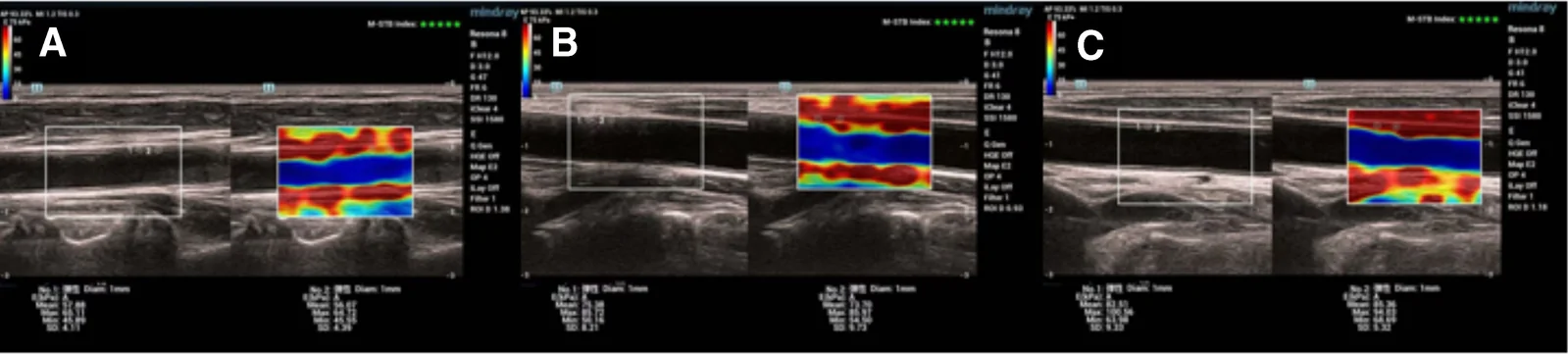
Carotid shear wave elastography images of patients in Group (A–C).

### 3.5. Correlation analysis

Correlation analysis indicated that MEmax, MEmean, and MEmin in each group were positively correlated with IMT (*R *= 0.312, 0.292, 0.304, *P *< .01), TC (*R *= 0.637, 0.658, 0.610, *P *< .01), TG (*R *= 0.571, 0.605, 0.547, *P *< .01), LDL-C (*R *= 0.505, 0.538, 0.485, *P *< .01) were all positively correlated. In contrast, MEmax, MEmean, and MEmin in each group were negatively correlated with HDL-C (*r *= −0.677, −0.666, −0.645, *P *< .01). (See Fig. 2).

**Figure 2.**
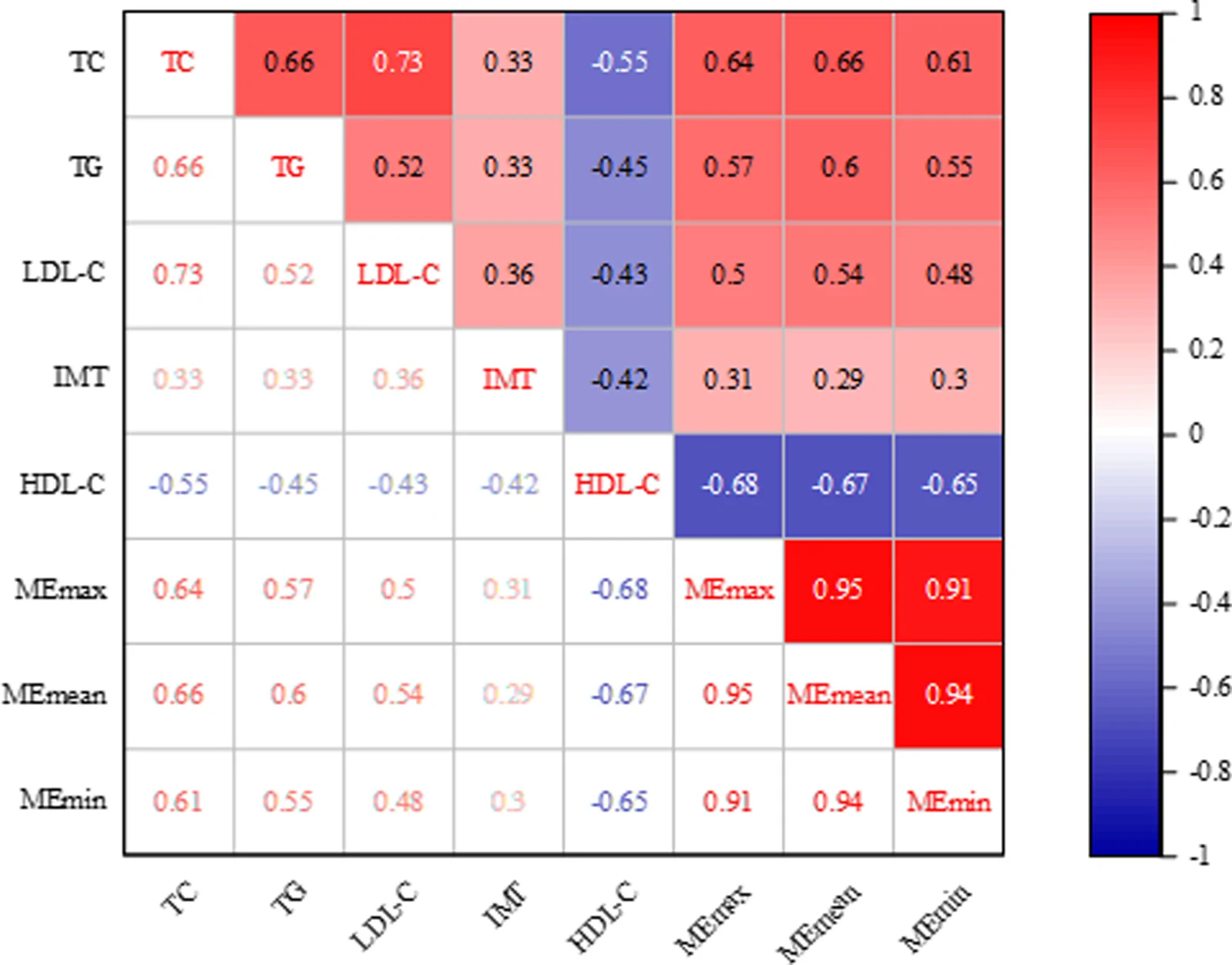
Heat map showing the correlation between IMT, TC, TG, LDL-C, HDL-C, and SWE parameters. TC = total cholesterol, TG = triglycerides, LDL-C = low-density lipoprotein cholesterol, HDL-C = high-density lipoprotein cholesterol, SWE = shear wave elastography.

Multivariable linear regression analysis showed that TC, TG, LDL-C, and HDL-C were significant influences on carotid artery elasticity in T2DM (all *P* < .05). (See Table 5).

**Table 5:** Relation between conventional risk factors and SWE-related parameters as determined by linear regression analysis.

	MEmax		MEmean		MEmin	
	*β*	*P*	*β*	*P*	*β*	*P*
Gander	0.006	.930	0.041	.538	0.023	.765
Age	0.047	.504	0.052	.441	0.049	.522
Duration	0.061	.386	0.026	.697	0.019	.802
Smoking	**0.220**	**.002**	**0.199**	**.003**	0.142	.063
BMI	0.040	.568	0.009	.896	0.011	.884
SBP	0.081	.347	0.019	.814	0.074	.433
HbA1c	0.028	.691	0.025	.715	0.043	.578
TC	**0.313**	**.003**	**0.339**	**.001**	**0.337**	**.004**
TG	**0.192**	**.016**	**0.233**	**.002**	0.159	.065
LDL-C	**0.170**	**.022**	**0.191**	**.007**	**0.207**	**.011**
HDL-C	**−0.399**	**.001**	**-0.341**	**.001**	**−0.331**	**.001**

*P* < .05 in bold.

### 3.6. Repetitive testing

The MEmax, MEmean, and MEmin values measured by SWE all had intraclass correlation coefficients values >0.90 within the observer, indicating good reproducibility in this study. (See Table 6 and Fig. 3).

**Table 6:** Assessment of intraobserver and interobserver reproducibility by calculating ICC.

	Intraobserver			Interobserver		
Parameter	ICC	95% Confidence Interval	*P*	ICC	95% Confidence Interval	*P*
MEmax	0.933	0.877–0.964	<.001	0.946	0.900–0.971	<.001
MEmean	0.922	0.857–0.958	<.001	0.926	0.865–0.960	<.001
MEmin	0.903	0.824–0.947	<.001	0.946	0.818–0.978	<.001

**Figure 3.**
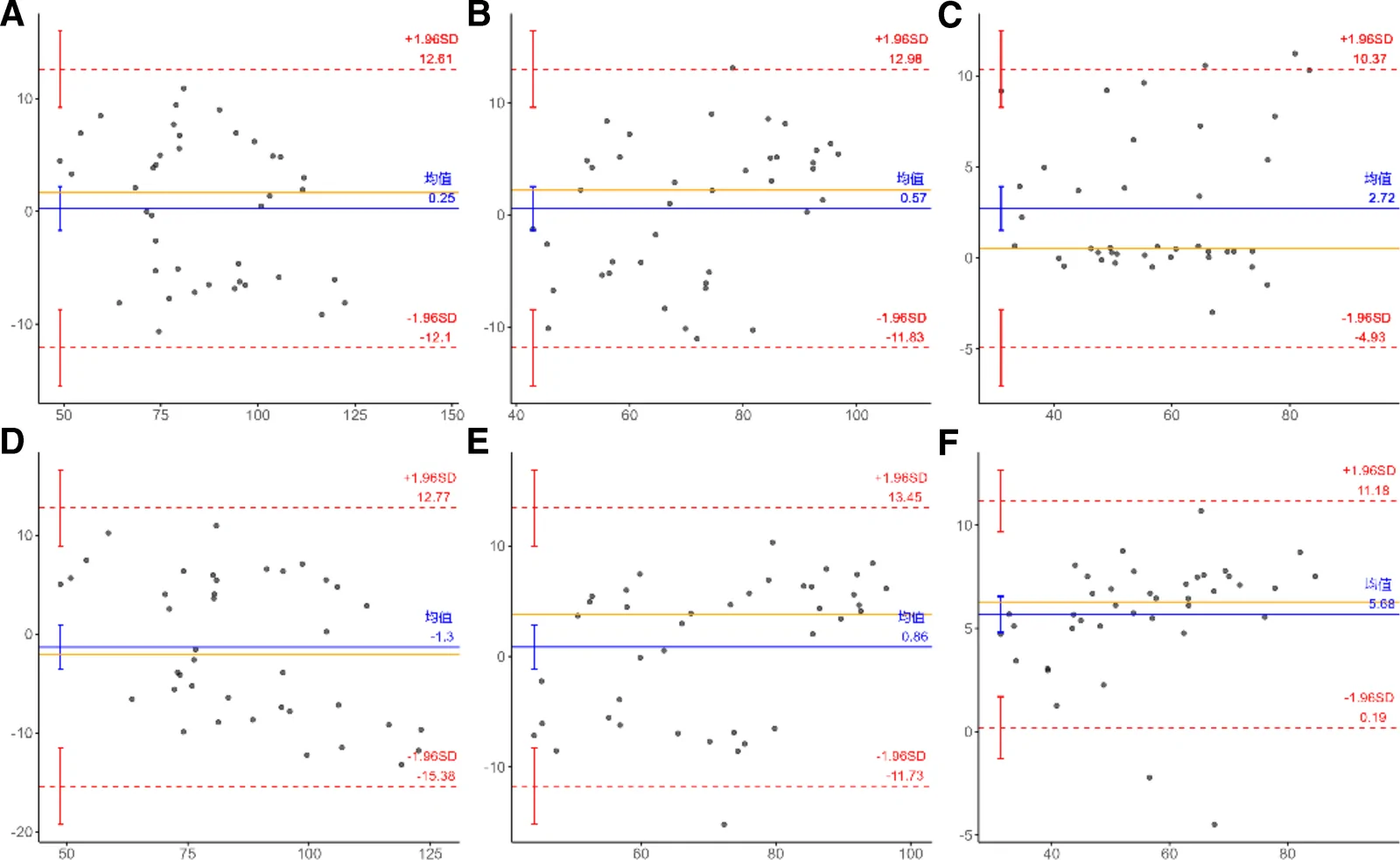
Bland-Altman plot of carotid SWE parameters. (A) MEmax interobserver variability. (B) MEmean interobserver variability. (C) MEmin interobserver variability. (D) MEmax intraobserver variability. (E) MEmean intraobserver variability. (F) MEmin intraobserver variability.

## 4. Discussion

Our findings demonstrated that HLD exacerbated carotid atherosclerosis in T2DM patients. Furthermore, SWE was able to sensitively detect early-stage changes in arterial stiffness, a result consistent with previous research.^[17,18]^

T2DM typically involves multiple systems and affects the functions of various organs. A decrease in carotid stiffness is a common complication associated with T2DM. Atherosclerosis serves as the primary pathological basis for carotid stiffness.^[3]^ Laurienne et al^[19]^ demonstrated that hyperglycemia induces hematopoietic stem cells and macrophages to undergo trained immunity, which significantly exacerbates atherosclerosis. HLD is commonly observed in T2DM patients, and its core mechanism involves multiple metabolic disorders caused by insulin resistance, ultimately leading to the abnormal accumulation of triglyceride-rich lipoproteins.^[20]^ The pathogenesis of atherosclerosis is driven by the accumulation of lipoproteins, particularly LDL, in the subendothelial layer or intima. Excessive cholesterol accumulation leads to the transformation of macrophages into foam cells, triggering an inflammatory response. Consequently, HLD promotes carotid atherosclerosis in patients with T2DM.^[21]^

The results of this experiment showed that the MEmax, MEmean, MEmin, CIMT, and *β* in Groups B and C were significantly higher than those in Group A. This can be attributed to 2 primary factors. First, the advanced glycation end-products in patients with T2DM interact with the receptor for AGEs expressed in smooth muscle cells, endothelial cells, and epithelial cells. This interaction leads to the cross-linking of collagen fibers, which disrupts the balance between collagen and elastin in the arterial wall, promoting arteriosclerosis. Second, impaired activation of nitric oxide synthase and increased production of superoxide reduce the bioavailability of nitric oxide, while decreased expression of vascular endothelial growth factor contributes to endothelial dysfunction.^[22,23]^ Among the various theories of atherosclerosis pathogenesis, the endothelial injury response hypothesis has garnered the most extensive support.^[24]^ Furthermore, the MEmax, MEmean, and MEmin values in Group C were significantly higher than those in Group B. This is primarily due to elevated triglyceride levels in plasma, which are rapidly hydrolyzed by lipoprotein lipase on the vascular wall, converting them into triglyceride-rich lipoprotein remnants and smaller lipoprotein particles. When the excessive production and/or impaired clearance of these remnants results in their retention in the bloodstream, they accumulate in the subendothelial space, thereby exacerbating the formation of atherosclerotic plaques.^[25]^ Katherine et al^[26]^ found that in diabetes and hyperlipidemia, immune regulatory factors promoted the progression of cardiovascular disease through metabolic mechanisms and epigenetic reprogramming dependent on bone marrow cells and their macrophage progeny. This process leads to the transcriptional activation of proinflammatory and atherogenic factors, thereby accelerating atherosclerosis, which is consistent with the findings of this study.

Conventional ultrasound is the primary method for evaluating early carotid atherosclerosis. However, it cannot assess carotid stiffness before morphological changes occur.^[27]^ The results of this study indicated that CIMT and *β* in Group C were slightly higher than those in Group B, but the differences were not statistically significant. This suggests that traditional ultrasound cannot quantitatively evaluate carotid artery stiffness in T2DM patients with HLD. In contrast, MEmax, MEmean, and MEmin in Group C were significantly higher than those in Group B, demonstrating that SWE detects carotid artery changes earlier than CIMT. In recent years, advanced ultrasound technologies have increasingly been used to study atherosclerotic carotid artery diseases, including contrast-enhanced ultrasound,^[28]^ ultrafast ultrasound imaging, and elastography.^[11,29]^ Related studies have shown^[30,31]^ that SWE directly quantifies local carotid artery wall stiffness by calculating the longitudinal elastic modulus. It is noninvasive, widely applicable, and considered a reliable method for evaluating carotid artery stiffness. In soft tissues, the shear wave velocity is approximately a thousand times slower than the velocity of conventional ultrasound waves, and shear wave attenuation occurs rapidly. However, shear waves propagate faster in stiffer tissues than in softer tissues, meaning that the velocity of shear waves is directly proportional to tissue stiffness, thereby providing a fixed measure of tissue hardness.^[32,33]^ Wang et al^[31]^ demonstrated that SWE is a useful predictive indicator for obstructive coronary artery disease in patients without carotid plaques. Guo et al^[16]^ used SWE to assess carotid stiffness in patients with metabolic syndrome, and the findings confirmed that SWE is a crucial instrument for determining these patients’ atherosclerosis risk.

FBG and HbA1c are reliable indicators of abnormal blood glucose levels. During the progression of plaque, specific molecules may diffuse into the plasma, providing information that can serve as alternative biomarkers. Lipid biomarkers, such as TC, TG, LDL-C, and HDL-C, are key risk factors and predictors of carotid atherosclerosis.^[34]^ In this study, the results of the biochemical indicators suggest that T2DM increases the risk of atherosclerosis, and that HLD in T2DM patients may synergistically exacerbate the progression of carotid atherosclerosis, reflecting the cumulative effects of metabolic disorders. Additionally, SWE parameters were positively correlated with CIMT in all 3 groups, demonstrating that SWE offers an innovative tool for the early detection and tracking of carotid atherosclerosis. In this study, SWE parameters were positively correlated with TC, TG, and LDL-C, and negatively correlated with HDL-C in all 3 groups, demonstrating that changes in vascular elastic modulus are closely associated with the severity of HLD in the progression of atherosclerosis. As key indicators for assessing vascular stiffness and predicting cardiovascular risk, the significance of these parameters increases as the disease progresses. Multivariable linear regression analysis was conducted to examine the relationship between SWE parameters and various clinical factors. The analysis revealed that SWE parameters were independently associated with key factors such as TC, TG, LDL-C, and HDL-C. These findings suggest that lipid dysregulation, particularly elevated TC, TG, and LDL-C, and reduced HDL-C, plays a significant role in influencing arterial stiffness in patients with T2DM.

This study has several limitations. First, due to the cross-sectional design, it cannot establish a causal relationship between HLD and carotid stiffness in patients with T2DM. Second, the relatively small, single-center cohort may limit the generalizability of the findings. Third, SWE itself has practical limitations, including operator dependence, motion artifacts from arterial pulsation and breathing, and so on. Finally, patients were treated with newer-generation diabetes medications and/or lipid-lowering therapies. However, the heterogeneity in treatment regimens limited the ability to assess their impact on carotid artery stiffness. Future prospective, multicenter studies with larger cohorts are warranted to confirm and extend our findings.

## 5. Conclusion

In conclusion, HLD exacerbates the severity of carotid atherosclerosis in patients with T2DM. SWE can accurately and rapidly evaluate the impact of hyperlipidemia on carotid stiffness in patients with T2DM.

## Acknowledgments

We thank the efforts and contributions of the reported patients and all the clinical staff in this study.

## Author contributions

**Validation:** Yuanyuan Liu, Xi Yang, Dan Lv, Guangsen Li.

**Investigation:** Xi Yang, Xuemeihui Ma.

**Conceptualization:** Xuemeihui Ma, Guangsen Li.

**Data curation:** Xuemeihui Ma, Dan Lv.

**Formal analysis:** Xuemeihui Ma.

**Project administration:** Dan Lv.

**Supervision:** Dan Lv, Guangsen Li.

**Writing – original draft:** Yuanyuan Liu.

**Writing – review & editing:** Yuanyuan Liu, Xi Yang.
